# Effects of feeding different postbiotic metabolite combinations produced by *Lactobacillus plantarum* strains on egg quality and production performance, faecal parameters and plasma cholesterol in laying hens

**DOI:** 10.1186/1746-6148-10-149

**Published:** 2014-07-05

**Authors:** Teck Chwen Loh, Di Wai Choe, Hooi Ling Foo, Awis Qurni Sazili, Mohd Hair Bejo

**Affiliations:** 1 Department of Animal Science, Faculty of Agriculture, Universiti Putra Malaysia, Serdang, Selangor 43400 UPM, Malaysia; 2 Institute of Tropical Agriculture, Universiti Putra Malaysia, Serdang, Selangor, Malaysia; 3 Department of Bioprocess Technology, Faculty of Biotechnology and Biomolecular Science, Universiti Putra Malaysia, Serdang, Selangor, Malaysia; 4 Institute of Bioscience, Universiti Putra Malaysia, Serdang, Selangor, Malaysia; 5 Department of Pathology and Microbiology, Faculty of Veterinary Medicine, Universiti Putra Malaysia, Serdang, Selangor, Malaysia

**Keywords:** Laying hen, *Lactobacillus plantarum*, Metabolite combination, Postbiotic

## Abstract

**Background:**

Probiotics are beneficial bacteria that are able to colonize the host digestive system, increasing the natural flora and preventing colonization of pathogenic organisms and thus, securing optimal utility of the feed. However, commercial probiotic often do not meet the expected standards and the viability of the efficacy of these strains remains questionable. Another major issue has been highlighted in relation to the application of antibiotic resistant probiotics, the antibiotic resistant gene can be transferred between organisms. Recently, postbiotic metabolites produced from microbes have been extensively studied as feed additive in order to substitute in-feed antibiotics.

**Results:**

No significant difference (*P > 0.05*) was found among the treatment groups on overall feed intake, egg weight, egg mass and feed conversion efficiency. COM456 had a significant reduction (*P < 0.05*) in faecal pH compared to the other groups at 28 weeks of age onwards. COM456 had significant higher (*P < 0.05*) level of lactic acid bacteria counts from 30 weeks of age onwards, followed by COM246 and COM345 at 32 and 34 weeks of age, respectively. Significant reduction of faecal *Enterobacteriaceae* (*P < 0.05*) were observed in COM246 and COM456 from 30 weeks of age onwards. The lowest levels (*P* < 0.05) of plasma and egg yolk cholesterol were observed in COM456, followed by COM345 and COM246. There was no significant difference in terms of yolk weight between the treatment groups. Significant higher (*P* < 0.05) content of C18:3, C20:2 and C22:6 were found in treatments supplemented with metabolite combinations as compared with the control group.

**Conclusions:**

The present study demonstrated the positive effects of metabolite combinations supplementation in laying hens. Increase in hen-day egg production was observed in all treatments supplemented with metabolite combinations. In addition, the metabolite combinations, COM456 had reduced the faecal pH and faecal *Enterobacteriaceae* population, improved the faecal lactic acid bacteria, reduced the plasma and yolk cholesterol and improved the faecal volatile fatty acids content. Postbiotic metabolite combinations can be used as an alternative feed additive to achieve high productivity and better animal health while reducing the use of conventional chemotherapeutic agents such as in-feed antimicrobials.

## Background

Layer poultry farms are forced to keep up with the latest trends in nutrition and management through modernization and industrialization. Efforts are made to increase the quantity and quality of egg yield through the manipulation of feed compositions and utilization of feed additives. The application of antibiotics as feed additives in poultry and pig production became virtually widespread since the discovery in the 1940’s [1]. However, the imprudent use of antimicrobials in human and animal health has led to an increase in the development of resistance of pathogens towards antimicrobial [2]. As the result, there has been a global effort to limit the sub-therapeutic use of feed antibiotics such as in the European Union and in the United States [3]. More efforts were made to explore alternative method to achieve high productivity and better animal health while reducing the use of conventional chemotherapeutic agents such as antimicrobials in the feed [4].

Probiotics are beneficial bacteria that are able to colonize the host digestive system, increasing the natural flora and preventing colonization of pathogenic organisms and thus, securing optimal utility of the feed [5]. However, commercial probiotic often do not meet the expected standards and the viability of the efficacy of these strains remains questionable [6,7]. Marteau and Shahanan [8] highlighted another major issue in relation to the application of probiotics where there is an occurrence of antibiotic resistance genes, especially those encoded by plasmids which can be transferred between organisms. Recently, metabolites produced from microbes or also known as postbiotic metabolites [9] has been extensively studied as feed additive in animal feedings [10,11]. Tsilingiri *et al*. [9] have demonstrated metabolites produced by probiotic bacterial have postbiotic activity. The postbiotic metabolite combinations have been demonstrated to be more effective than single postbiotic metabolite in broiler [12] and pigs [13] feedings. Postbioitc metabolites of *L. plantarum* strains employed in current study are intermediate and final products in metabolism of probiotic bacterial*,* which contain mainly lactic acid, acetic acids and bacteriocin inhibitory activity [14,15]. A novel combinations of 2 classes of bacteriocin genes: plantaricin EF and plantaricin W was successfully amplified from the *L. plantarum* strains by Moghadam *et al*. [16]. Furthermore, Thanh *et al.*[17] reported that different combinations of postbiotic metabolites of *L. plantarum* strains had different inhibitory spectrum against *Escherichia coli, Listeria monocytogenes, Salmonella typhimurium* and vancomycin resistant enterococci. Similarly, Choe *et al.*[15] used six different strains of *L. plantarum* to produce postibiotic metabolites in order to study inhibitory property of their combinations. Three combinations of postbiotic metabolites from three *L. plantarum* strains contributed higher inhibitory activity against *Pediococcus acidilactici* as compared with single strain of postbiotic metabolite. Recently, these metabolites have been shown to have many beneficial probiotic effects on animal growth performances and particularly in the gut health when use as additive in animal diet [12,13]. However, the additive effects of different postbiotic metabolite combinations on the egg production performance, Haugh unit, faecal pH, volatile fatty acids and microflora (lactic acid bacteria and *Enterobacteriaceae*) counts, blood plasma cholesterol, yolk cholesterol and fatty acid composition of laying hens have not been reported elsewhere. Therefore, this study was conducted to evaluate the additive effects of different postbiotic metabolite combinations produced from *L. plantarum* strains in the diet of laying hens.

## Methods

### Bacteria source, experimental animals and diets

Five strains of *L. plantarum* (TL1, RS5, RG14, RG11 and RI11) were used in this study. These strains were previously isolated from Malaysian fermented food by the Laboratory of Industrial Biotechnology, Department of Bioprocess Technology, Faculty of Biotechnology and Biomolecular Sciences, Universiti Putra Malaysia.

A total of 200 Lohman Brown layers, aged 23 weeks, were randomly assigned to four numerically equal groups, with 25 replicates per treatment, two birds per cage with the size of 55 × 50 × 45 cm^3^ (width × depth × height). The feeding period lasted 12 weeks, commencing when the layers were 23 weeks of age and ending when they were 35 weeks of age, with addition of 1 week for feed adaptation. Water and basal diet were supplied *ad libitum*. The dietary treatments (Table 1) consisted of: (i) basal diet without supplementation of metabolites (control); (ii) basal diet supplemented with 0.6% COM246 metabolites (TL1, RI11 and RG11); (iii) basal diet supplemented with 0.6% COM345 metabolites (RS5, RI11 and RG14); and (iv) basal diet supplemented with 0.6% COM456 metabolites (RI11, RG14 and RG11). All the metabolite combinations were added into the feed in liquid form (v/w). The metabolite combinations were selected and prepared based on the results obtained by Thu *et al*. [14] as different metabolite combinations have different inhibitory activity and levels of organic acids such as lactic and acetic acids. The metabolite combinations used in this study were prepared and stored separately and were mixed at equal ratios before being added into the feed.

**Table 1:** Ingredients and composition of experimental basal diet

**Ingredients (%)**	**Control**	**0.6%**	**0.6%**	**0.6%**
		**COM246**^**3**^	**COM345**^**4**^	**COM456**^**5**^
Yellow corn	58.20	58.20	58.20	58.20
Soybean meal	24.10	24.100	24.10	24.10
Wheat	3.80	3.20	3.20	3.20
Crude palm oil	1.55	1.55	1.55	1.55
L-lysine	0.06	0.06	0.06	0.06
DL-methionine	0.16	0.16	0.16	0.16
MDCP	2.50	2.50	2.50	2.50
Limestone	8.00	8.00	8.00	8.00
Common salt	0.50	0.50	0.50	0.50
Vitamin premix^1^	0.07	0.07	0.07	0.07
Mineral premix^2^	0.06	0.06	0.06	0.06
Choline chloride	1.00	1.00	1.00	1.00
COM metabolite	-	0.60	0.60	0.60
Total	100	100	100	100
Calculated values				
ME(Cal/kg)	2805.32	2804.48	2806.17	2805.73
Crude protein, %	16.83	16.86	16.81	16.85
Calcium, %	3.70	3.70	3.70	3.71
Avai. Phophorus, %	0.45	0.45	0.45	0.45

^1^Vitamin premix provided per kilogram of diet: retinol 3.00 mg; cholecalciferol 0.06 mg; α-tocopherol 15.00 mg; thiamine 1.20 mg; riboflavin 4.00 mg; pantothenic acid 8.00 mg; pyridoxine 2.00 mg; niacin 30.00 mg; cobalamin 0.02 mg; folic acid 0.50 mg; biotin 0.03 mg; menadione 3.00 mg.

^2^Mineral premix provided per kilogram of diet: manganese 100.0 mg; copper 8.0 mg; iodine 0.8 mg; cobalt 0.25 mg; selenium 0.3 mg; zinc 80.0 mg; iron 40.0 mg.

^3^Com246 is a combination of 3 strains TL1, RI11 and RG11.

^4^Com345 is a combination of 3 strains RS5, RI11and RG14.

^5^Com456 is a combination of 3 strains RI11, RG14 and RG11.

### Egg production performance

Feed intake was measured by subtracting the balance of feed from the quantity originally supplied to the laying hens. Eggs from individual cages were collected daily and weighed. The hen-day egg production and feed efficiency were calculated as the rate of production per hen per day and feed intake/egg mass.

Determination of the Haugh unit was conducted using Egg Analyzer™ (SANOVO, Denmark). After weighing, the egg was broken and placed on the measuring plate. The yolk colour, height, thickness of egg white and the Haugh unit were automatically measured, calculated and recorded. The formula for calculating the Haugh unit:

$$\mathrm{Haugh}\;\mathrm{Unit}=\;100\;*\;\mathrm{Lo}g_{10}\left(h-\;1.7w^{0.37}+\;7.6\right)$$

h = height of the albumen (mm); w = weight of egg in grams (g)

### Faecal pH, faecal lactic acid bacteria and *Enterobacteriaceae*

Twenty five fresh faecal samples per treatment were collected fortnightly in the morning, using a sterile plastic bag from every cage, and stored in the chiller prior to laboratory analysis. One gram of fresh faecal sample was mixed homogeneously with 9 ml of deionised distilled water in a sterile tube. The pH was measured using a pH meter (Mettler-Toledo, England) calibrated using a buffer solution (Merck, KGaA, Darmstadt, Germany) at pH 4 and 7. The faecal microflora (lactic acid bacteria and *Enterobacteriaceae*) counts were conducted according to the method as described by Foo *et al*. [18] and Loh *et al*. [12]. Enumerations of lactic acid bacteria were carried out on Lactobacillus agar DE Man, ROGOSA and SHARPE (MRS-agar, Merck, KgaA, Darmstadt, Germany). The plates were incubated anaerobically at 30°C for 48 h. ENT were spread and enumerated on Eosin-methylene-blue lactose sucrose agar plates (EMB-agar, Merck, KgaA, Darmstadt, Germany) and incubated aerobically for 24 h at 37°C. The number of colony forming units (CFU) was expressed as the base 10 logarithm of CFU (log_10_ CFU) per gram. All analyses were conducted in triplicate.

### Determination of cholesterol and fatty acids in plasma and egg yolk

At the end of the study, twelve birds from each treatment were randomly selected for blood samples. The blood was collected via jugular vein puncture into tubes containing EDTA (vacutainer, USA) for plasma cholesterol analysis. Plasma was prepared by centrifuging the blood at 1000 *g* for 10 min. The plasma was then transferred into a microcentrifuge tube using a Pasteur pipette and stored at −20°C until further analyses. The plasma was allocated for total cholesterol analyses as described by Choe *et al*. [19].

Twelve eggs per treatment were randomly selected for the analysis of yolk cholesterol and fatty acid contents. Egg yolk was obtained by discarded albumen. The egg yolk was homogenized gently without foaming for 30 s by hand to minimize possible fatty acid oxidation. About 0.3-0.5 g of liquid egg yolk was used for fatty acid analyses. The yolk sample was homogenized in 40 mL of chloroform-methanol (2:1, v/v) in a 50 mL stoppered ground-glass extraction tube. The tube was flushed with purified nitrogen gas, stoppered and then shaken vigorously before being allowed to stand for 12 h. The total fatty acid extractions hereafter were performed as described by Loh *et al*. [20]. The remaining liquid yolk was subjected to total cholesterol extraction immediately following the procedure for cholesterol determination as described by Choe *et al*. [19].

### Faecal volatile fatty acid determination

At the end of the study, twenty five fresh faecal samples per treatment were collected for the volatile fatty acid determination. The determination was done using GLC method as described by Thanh *et al.*[21]. One gram of faeces was added into a rest tube containing one mL of 24% (v/v) metaphosphoric acid which was diluted in sulfuric acid 3 N. The acidic mixture was left at room temperature for overnight and centrifuged at 10 000 G for 20 min at four °C. Finally, the supernatant was collected into a 2 mL screw-capped vial (Kimble Glass Co., USA). The internal standard (20 mM 4-methyl-butyric acid, Sigma Aldrich Chemical GmbH, Germany) was added and the samples were kept at −20°C until analysis using GC. The volatile fatty acid separation was based on a Quadrex 007 series capillary column (15 m, 0.32 mm ID, 0.25 μm film thickness) in a 6890 N Hewlett-Packard GC. The injector and detector temperature were programmed at 230°C and the column temperature was set at the 200°C.

### Statistical analysis

The data were analyzed by one-way analysis of variance (ANOVA) using the GLM procedure by Statistical Analysis System [22]. Duncan’s Multiple Range Test System was used to compare the significant difference between the treatments at *P* < 0.05. The results of statistical analysis were presented as mean ± standard error of the mean (SEM).

## Results

### Egg production performance

There was no significant difference (*P > 0.05*) among the treatment groups on overall feed intake, egg weight, egg mass and feed conversion efficiency (Table 2). However, a significantly higher (*P < 0.05*) overall hen-day egg production was observed in all treatment groups supplemented with metabolite combination as compared with the control. The highest egg production was seen in COM456. The Haugh unit of eggs are shown in Table 3. There was no significant difference (*P* > 0.05) in Haugh unit along the experimental period. The highest Haugh unit was observed in 30 and 32 weeks of age. In general, all of the eggs sampled from 24 to 32 weeks of age were categorized as Grade A based on USDA egg-grading standard.

**Table 2:** Feed intake, hen-day egg production, egg weight, egg mass and feed conversion efficiency following treatments supplemented with different postbiotic metabolite combinations

		**Treatments**		
	**Control**	**COM246**	**COM345**	**COM456**
Hen-day egg production (%)	83.07 ± 1.02^b^	85.21 ± 0.76^a^	85.10 ± 0.61^a^	86.43 ± 1.08^a^
Feed intake (g/hen/day)	98.88 ± 2.24	99.24 ± 2.81	98.97 ± 3.21	99.13 ± 2.67
Egg weight (g/egg)	58.72 ± 1.71	57.63 ± 1.77	57.42 ± 1.64	56.81 ± 1.87
Egg mass (g/hen/day)	48.78 ± 0.46	49.00 ± 0.52	48.86 ± 0.55	49.08 ± 0.65
Feed conversion efficiency	2.02 ± 0.13	2.03 ± 0.16	2.02 ± 0.11	2.02 ± 0.12

^1 a-b^Means in the same row not sharing a common superscript are significantly *P* < 0.05).

^2^SEM: Data are means of 25 cages of 2 hens per cage per treatmen.t.

**Table 3:** **Haugh unit of eggs, faecal pH, lactic acid bacteria and***Enterobacteriaceae***counts following treatments supplemented with different postbiotic metabolite combinations**

		**Treatments**		
**Weeks**	**Control**	**COM246**	**COM345**	**COM456**
Haugh unit				
24	68.89 ± 0.81	69.24 ± 0.72	68.26 ± 0.85	68.62 ± 0.78
26	67.13 ± 0.93	67.42 ± 0.94	67.34 ± 0.86	66.94 ± 0.79
28	67.77 ± 0.93	67.80 ± 0.95	67.32 ± 0.86	66.75 ± 0.75
30	73.32 ± 0.87	72.97 ± 0.66	73.36 ± 0.74	72.82 ± 0.60
32	73.50 ± 0.82	73.29 ± 0.63	73.17 ± 0.87	72.92 ± 0.71
pH				
24	7.04 ± 0.03	7.05 ± 0.04	7.01 ± 0.05	7.03 ± 0.03
26	6.98 ± 0.04	7.02 ± 0.03	6.99 ± 0.03	7.01 ± 0.04
28	7.02 ± 0.04^a^	6.98 ± 0.04^a^	7.00 ± 0.02^a^	6.82 ± 0.05^b^
30	6.98 ± 0.05^a^	6.86 ± 0.04^b^	6.87 ± 0.05^b^	6.78 ± 0.03^c^
32	6.94 ± 0.06^a^	6.65 ± 0.05^b^	6.54 ± 0.04^c^	6.52 ± 0.05^c^
Lactic acid bacteria counts (log CFU/g)				
24	6.45 ± 0.03	6.48 ± 0.06	6.42 ± 0.06	6.41 ± 0.07
26	6.39 ± 0.11	6.32 ± 0.07	6.31 ± 0.12	6.34 ± 0.08
28	6.48 ± 0.12	6.57 ± 0.10	6.53 ± 0.11	6.60 ± 0.19
30	6.56 ± 0.18^b^	6.61 ± 0.16^b^	6.68 ± 0.14^b^	6.92 ± 0.11^a^
32	6.62 ± 0.16^c^	6.91 ± 0.18^b^	6.68 ± 0.13^c^	7.28 ± 0.10^a^
34	6.45 ± 0.21^c^	6.88 ± 0.18^b^	6.90 ± 0.10^b^	7.32 ± 0.12^a^
*Enterobacteriaceae* counts (log CFU/g)				
24	7.43 ± 0.08	7.47 ± 0.06	7.51 ± 0.05	7.56 ± 0.09
26	7.50 ± 0.05	7.52 ± 0.06	7.51 ± 0.06	7.46 ± 0.07
28	7.38 ± 0.10	7.41 ± 0.18	7.32 ± 0.14	7.25 ± 0.12
30	7.16 ± 0.08^a^	6.89 ± 0.09^b^	6.78 ± 0.10^b^	6.83 ± 0.07^b^
32	6.92 ± 0.16^a^	6.33 ± 0.11^c^	6.55 ± 0.08^b^	6.31 ± 0.12^c^

^1^Means in the same row not sharing a common superscript are significantly different (*P* < 0.05).

^2^SEM: Data are means of 25 cages of 2 hens per cage per treatment.

### Faecal pH, faecal lactic acid bacteria and *Enterobacteriaceae*

The faecal pH for various treatment groups is shown in Table 3. There was no significant difference (*P > 0.05*) in faecal pH among the treatment groups at the first 4 weeks of sampling. However, COM456 started to show significant reduction (*P < 0.05*) in faecal pH compared to the other groups at 28 weeks of age onwards. COM345 and COM246 started to exhibit significant (*P < 0.05)* effect in reducing faecal pH compared to the control group from 30 weeks of age onwards.

Faecal lactic acid bacteria and *Enterobacteriaceae* counts are presented in Tables 3. There was no significant difference (*P > 0.05*) in lactic acid bacteria count among the dietary treatments at 24 to 28 weeks of sampling. However, COM456 started to show significant (*P < 0.05*) higher level of lactic acid bacteria counts from 30 weeks of age onwards. COM246 showed significant (*P < 0.05*) increase in faecal lactic acid bacteria counts on 32 weeks of age onwards and followed by COM345 at 34 weeks of age. In contrast, significant reduction of faecal *Enterobacteriaceae* (*P < 0.05*) were observed in all groups supplemented with metabolite combinations from 30 weeks of age onwards. COM246 and COM456 have the lowest (*P < 0.05*) *Enterobacteriaceae* count among the treatments.

### Cholesterol **and fatty acid profiles** in plasma and yolk

The plasma and yolk cholesterol compositions are presented in Table 4. At the end of the experiment, all treatment groups supplemented with metabolite combination had significant reductions (*P* < 0.05) in plasma cholesterol. The lowest levels (*P* < 0.05) of plasma cholesterol were observed in COM456, followed by COM345 and COM246. As for the yolk cholesterol analysis, there was no significant difference in terms of yolk weight between the treatment groups. However, COM456 produced lowest level of yolk cholesterol (197.22 mg/yolk). This was followed by COM246 and COM345. The control group has the highest level of yolk cholesterol. The yolk fatty acid compositions are as presented in Table 4. A total of 12 fatty acids were quantified including: C14:0 (Mystiric acid), C16:0 (Palmitic acid), C16:1 (Palmitoleic acid, C18:0 (Stearic acid), C18:1 (Oleic acid), C18:2 (Linoleic acid), C18:3 (Linolenic acid), C20:1 (Eicosenoic acid), C20:2 (Eicosadienoic acid), C20:4 (Arachidonic acid), C22:5 (Docosapentaenoic acid) and C22:6 (Docosahexaenoic acid). There was no significant difference (*P* > 0.05) in total fatty acids among the dietary treatments. However, higher (*P* < 0.05) content of C18:3, C20:2 and C22:6 were found in treatments supplemented with metabolite combinations as compared with the control group. The significantly higher (*P* < 0.05) level of linolenic acid and docosahexaenoic acid and lower level of eicosadienoic acid had contributed to the reduction of n-6: n-3 ratio.

**Table 4:** Plasma and egg yolk cholesterol, and fatty acid profiles following treatments supplemented with different postbiotic metabolite combinations

		**Treatments**		
**Parameters**	**Control**	**COM246**	**COM345**	**COM456**
Plasma cholesterol (mg/dL)	171.83 ± 2.90^a^	155.54 ± 1.69^b^	157.91 ± 1.48^b^	145.24 ± 1.86^c^
Yolk weight(g)	16.41 ± 0.02	16.40 ± 0.03	16.41 ± 0.02	16.40 ± 0.03
Yolk cholesterol (mg/100 g)	1276.00 ± 6.33^a^	1226.00 ± 9.21^b^	1233.00 ± 8.87^b^	1202.00 ± 6.04^c^
Yolk cholesterol (mg/yolk)	209.65 ± 1.63^a^	201.84 ± 1.06^b^	202.43 ± 1.83^b^	197.22 ± 1.21^c^
Egg yolk fatty acid (%)				
Mystiric acid (C14:0)	0.49 ± 0.03	0.49 ± 0.04	0.48 ± 0.02	0.46 ± 0.05
Palmitic acid (C16:0)	23.99 ± 0.51	23.79 ± 0.48	23.61 ± 0.55	23.66 ± 0.61
Palmitoleic acid (C16:1 n-9)	3.52 ± 0.42	3.39 ± 0.51	3.47 ± 0.44	3.40 ± 0.40
Stearic acid (C18:0)	8.41 ± 0.14	8.43 ± 0.17	8.49 ± 0.23	8.45 ± 0.16
Oleic acid (C18:1 n-9)	41.03 ± 1.15	41.16 ± 1.28	41.09 ± 1.49	41.40 ± 1.60
Linoleic acid (C18:2 n-6)	16.89 ± 0.87	16.88 ± 0.62	16.92 ± 0.71	16.48 ± 0.89
Linolenic acid (C18:3 n-3)	0.34 ± 0.02^b^	0.40 ± 0.02^a^	0.33 ± 0.04^b^	0.42 ± 0.02^a^
Eicosenoic acid (C20:1 n-9)	0.05 ± 0.01	0.06 ± 0.01	0.06 ± 0.01	0.06 ± 0.01
Eicosadienoic acidC20:2 n-6	0.31 ± 0.03^b^	0.32 ± 0.02^b^	0.38 ± 0.02^a^	0.31 ± 0.03^b^
Arachidonic acid (C20:4 n-6)	2.91 ± 0.33	2.77 ± 0.43	2.82 ± 0.35	2.77 ± 0.38
Docosapentaenoic acid (C22:5 n-3)	1.07 ± 0.13	0.97 ± 0.14	1.07 ± 0.12	1.00 ± 0.11
Docosahexaenoic acid (C22:6 n-3)	0.98 ± 0.08^c^	1.34 ± 0.09^b^	1.28 ± 0.06^b^	1.59 ± 0.11^a^
Total fatty acids	14787.10 ± 125.40	14811.90 ± 135.60	14852.8 ± 146.20	14799.90 ± 156.80
Saturated fatty acids (%)	32.89 ± 1.23	32.70 ± 1.44	32.58 ± 1.32	32.56 ± 1.08
Unsaturated fatty acids (%)	67.10 ± 1.29	67.29 ± 1.56	67.40 ± 1.87	67.44 ± 1.66
n-6:n-3 ratio	8.37 ± 0.27^c^	7.36 ± 0.32^b^	7.51 ± 0.27^b^	6.53 ± 0.28^a^
Unsaturated fatty acid: Saturated fatty acid ratio	2.03 ± 0.14	2.06 ± 0.13	2.07 ± 0.12	2.07 ± 0.13
Polyunsaturated fatty acid: Saturated fatty acid ratio	0.68 ± 0.04	0.69 ± 0.03	0.70 ± 0.04	0.69 ± 0.05

^1 a-c^Means in the same row not sharing a common superscript are significantly different (*P* < 0.05); ^2^SEM: Data are means of 12 birds per treatment.

### Faecal volatile fatty acid concentrations

The effects of different dietary treatments on faecal volatile fatty acid are as presented in Table 5. The concentration of total volatile fatty acid increased significantly (*P* < 0.05) in the faeces of layers fed with metabolite combinations. Acetic acid, iso-butyric acid and iso-valeric acid were not significantly affected (*P* > 0.05). However, higher composition (*P* < 0.05) of propionic and butyric acid were observed in treatments supplemented with metabolite combinations. Acetic acid was the major volatile fatty acid found in faecal samples. However, valeric acid was not detected in the present study.

**Table 5:** Faecal volatile fatty acids following treatments supplemented with different postbiotic metabolite combinations

		**Treatments**		
**Parameters**	**Control**	**COM246**	**COM345**	**COM456**
Total volatile fatty acids (mM)	61.48 ± 2.13^b^	68.38 ± 1.83^a^	68.32 ± 1.48^a^	68.95 ± 1.61^a^
Acetic	40 .11 ± 1.78	43.42 ± 1.28	43.65 ± 2.87	43.98 ± 1.63
Priopionic	8.52 ± 0.50^b^	9.62 ± 0.44^a^	9.78 ± 0.61^a^	9.61 ± 0.52^a^
Butyric	6.47 ± 0.53^b^	8.83 ± 0.67^a^	8.38 ± 0.75^a^	8.67 ± 0.63^a^
iso-butyric	1.32 ± 0.10	1.42 ± 0.08	1.33 ± 0.12	1.44 ± 0.09
iso-valeric	5.03 ± 0.18	5.06 ± 0.12	5.13 ± 0.14	5.15 ± 0.13
pH value	6.94 ± 0.06^a^	6.65 ± 0.05^b^	6.54 ± 0.04^c^	6.52 ± 0.05^c^

^1 a-c^Means in the same row not sharing a common superscript are significantly different (*P* < 0.05).

^2^SEM: Data are means of 25 cages of 2 hens per cage per treatment.

## Discussion

### Egg production performance

The feed intake, egg weight, egg mass and feed conversion efficiency were not significantly different (*P* > 0.05) among the treatment groups. However, higher overall hen-day egg production was observed in the treatment group supplemented with 0.6% of COM456, compared with other groups. These results were coincided with Panda *et al*. [23] which indicated similar attributes, when volatile fatty acid cultures were added to the diets of laying hens. Another study by Xu *et al.*[24] showed improvement not only in egg production but in egg mass as well. In contrast, Mahdavi *et al.*[25] reported that the inclusion of lactic acid bacteria cultures did not affect any egg production parameters. The variations in the results were most probably due to the difference in bacteria strains, concentration and form which was used [25]. The supplementation of metabolite in the diets of laying hens may exert different effects compared to live probiotic cultures.

Haugh unit is a measurement of the internal quality of an egg, which is correlated to its weight and albumin height. In this study, the Haugh unit was not affected by the supplementation of metabolite combinations. This is in agreement with studies conducted by Mahdavi *et al.*[25] and Aghaii *et al.*[26] where supplementation of probiotic in the diet of laying hens did not affect the albumin quality of the egg. Various studies have shown that the albumin quality will only be affected by factors such as bird strains, storage time [27], nutrition and diseases [1].

### Faecal pH, faecal lactic acid bacteria and *Enterobacteriaceae*

Significant reduction of faecal pH was observed in all treatments supplemented with metabolite combinations. The finding could be due to the presence of organic acids such as lactate and acetate in the metabolite which act as an acidifying agent [14]. Previous research had reported similar finding when *Lactobacillus* metabolite was added into diet of broilers [12,21] and pigs [13].

The present study demonstrated the effect of metabolite combination supplementation in reducing (*P* < 0.05) faecal *Enterobacteriaceae* and increasing (*P* ≤ 0.05) lactic acid bacteria counts. This is due to the presence of different levels of organic acids and bacteriocins in the metabolite. Short chain acids such as acetate, propionate and butyrate and carboxylic acid such as lactate are associated with potent antimicrobial activity [28,29]. This could be due to undissociated form of organic acids enters into microbial cell via carrier-mediated transport mechanism. In the cell, the acids releases proton which resulting in a decrease of intracellular pH. This causes inhibition of vital microbial enzyme synthesis as remaining energy is used to release protons leading to accumulation of acid anions. As lactic acid bacteria is able to grow well in relatively low pH environment, they are able to tolerate well with the condition as compared with *Enterobacteriaceae*. Thu *et al.*[13] reported similar observations following addition of metabolite combinations into the diet of piglets.

Bacteriocin is an antimicrobial pepetide produced by various genera of bacteria which is effective against bacteria of closely related strains [30]. However, the presence of other antimicrobial compounds in the metabolite such as organic acids could potentiate the inhibitory spectrum of bacteriocins. In addition, Luders *et al.*[31] and Thanh *et al.*[17] showed stronger inhibitory effect against pathogens such as *E. coli* when bacteriocins from different strains of bacteriocin producing bacteria were mixed. Therefore, the metabolite added into the diet of laying hens was able to provide more conducive environment for the growth of lactic acid bacteria while suppressing pathogenic bacteria such as coliforms at the same time.

### Cholesterol and yolk fatty acid profiles in plasma and yolk

The plasma cholesterol decreased significantly in the treatments supplemented with metabolite as compared with the control group. The possible reasons of reduction were due to the increase activity of lactic acid bacteria in the gut system. Lee *et al*. [32] clarified that higher population of lactic acid bacteria could (1) inhibits synthesis of cholesterol enzymes in the host, (2) assists the elimination of cholesterol in the faeces, (3) utilizes circulating cholesterol for the synthesis of bacterial cell wall and thus reducing the cholesterol level and (4) enhancing bile salts deconjugation and excretion which signals the host to utilize more cholesterol to synthesize new bile salts. Similar findings were noted when metabolite combinations were added into the diets of rats [10,33], broilers [34] and pigs [35].

Treatments supplemented with metabolite combinations have lower level of yolk cholesterol as compared with the control group. Panda *et al*. [23] observed similar result when probiotic cultures containing beneficial metabolites were added into the diet of laying hens while Mahdavi *et al*. [25] and Li *et al*. [36] reported a trend of reduction not only in yolk cholesterol, but a decrease in serum cholesterol as well. The possible explanation could be due to the lower level of cholesterol present in the blood circulation which makes it less available to be deposited into the egg yolk. Cholesterol is transported in the blood circulation by very low density lipoprotein to the ovarian membrane and lastly deposited into the developing yolk. The n-6: n-3 ratio and polyunsaturated fatty acid: saturated fatty acid are the two common parameters used to determine the nutritional quality of the lipid fractions in the food products [37]. In the present study, the n-6: n-3 ratio in treatments supplemented with metabolite combination was significantly lower as compared to the control group. This was contributed by higher concentration of total n-3 (linolenic and docosahexaenoic acid) and reduction in total n-6 (eicosadienoic acid) fatty acids. Linolenic and docosahexaenoic acids are members of essential fatty acids which exerts various health benefits upon consumption. In human health, diets of high linolenic acid have been associated with prevention of coronary heart diseases [38] while regular docosahexaenoic acid consumption may inhibit growth of cancer cells [39]. Leshkanich and Noble [40] stated that the egg yolk fatty acid composition can be manipulated the most through the changes of fatty acids in the diet. Likewise, Thu *et al*. [35] also reported similar finding when *Lactobacillus* metabolite was added in the diet of weaning pigs.

### Faecal volatile fatty acid concentrations

The metabolite produced from *Lactobacillus* contains beneficial compounds (bacteriocin and organic acids) which provide favorable condition for the growth of lactic acid bacteria. Higher population of lactic acid bacteria would implicate beneficial effects to the host such as inhibiting over population of pathogenic bacteria, modulation of the immune response and production of volatile fatty acids [41]. Volatile fatty acids are among the key products which are known for their bacteriostatic effect against wide range of pathogenic bacteria [42]. In this study, the total volatile fatty acid concentrations were higher in treatments supplemented with metabolite combination which is in agreement with Thanh *et al*. [21] and Thu *et al*. [13]. This could be due to the increase of lactic acid bacteria population in the gut system. Among the volatile fatty acid identified in this study, the concentration of acetic acid was the highest. Immerseel *et al*. [43] reported that one of the major end products from bacterial fermentation in the gut is acetic acid.

## Conclusions

The present study demonstrated the positive effects of postbiotic metabolite combinations supplementation in laying hens. Increase in hen-day egg production was observed in all treatments supplemented with metabolite combinations. In addition, the metabolite combinations, COM456 had reduced the faecal pH and faecal *Enterobacteriaceae* population, improved the faecal lactic acid bacteria, reduced the plasma and yolk cholesterol and improved the faecal volatile fatty acids content. On the contrary, no significant effect was observed in the albumin quality. Postbiotic metabolite combinations can be an alternative feed additive to achieve high productivity and better animal health while reducing the use of conventional chemotherapeutic agents such as in-feed antimicrobials.

## Competing interest

The authors declare that they have no competing interests.

## Authors’ contributions

FHL and LTC provided probiotic strains and method to produce postbiotic metabolite. CDW, LTC, FHL and AQS participated in the whole design of the study and performed the statistical analysis. CDW, FHL, LTC, AQS and MHB carried out laboratory analyses and contributed to the preparation of the manuscript. All authors read and approved the final manuscript.

## References

[B1] Toussant MJ Latshaw JD Ovomucin contents and composition in chicken eggs with different interior quality J Sci Food Agric 1999 79 1666 1670

[B2] Williams RJ Heymann DL Containment of antibiotic resistance Science 1998 279 1153 1154 9508688 10.1126/science.279.5354.1153

[B3] Ratcliff J Antibiotic Bans: A European Perspective Proceedings of the AFMA Symposium on: Improving Animal Performance Through Nutrition 2000 2000 Pretoria, South Africa: AFMA 128

[B4] Turner JL Pas S Dritz S Minton JE Review: **Alternatives to conventional antimicrobials in swine diets** The Professional Animal Scientist 2002 17 217 226

[B5] Vanbelle M Teller E Focant M Probiotics in animal nutrition: A review Berlin Archive of Animal Nutrition 1990 7 543 567 10.1080/17450399009428406 2264760

[B6] Weese JS Microbiological evaluation of commercial probiotics J Am Vet Med Assoc 2002 220 794 797 11918274 10.2460/javma.2002.220.794

[B7] Fasoli S Marzotto M Rizotti L Rossi F Dellaglio F Torriani S Bacterial composition of commercial probiotic products as evaluated by PCR-DGGE analysis Int J Food Microbiol 2003 82 59 70 12505460 10.1016/s0168-1605(02)00259-3

[B8] Marteau P Shanahan F Basic aspects and pharmacology of probiotics: An overview of pharmacokinetics, mechanisms of action and side effects Best Practice and Research in Clinical Gastroenterology 2003 17 725 740 10.1016/s1521-6918(03)00055-6 14507584

[B9] Tsilingiri K Barbosa T Penna G Caprioli F Sonzogni A Viale G Rescigno M Probiotic and postbiotic activity in health and disease: comparison on a novel polarised ex-vivo organ culture model Gut 2012 61 1007 1015 22301383 10.1136/gutjnl-2011-300971

[B10] Loh TC Lee TM Foo HL Law FL Rajion MA Growth performance and faecal microflora of rats offered with metabolites from lactic acid bacteria in drinking water J Appl Anim Res 2008 34 61 64

[B11] Loh TC Law FL Foo HL Goh YM Zulkifli I Effects of feeding fermented fish on egg cholesterol content in hens Anim Sci J 2009 80 27 33 20163464 10.1111/j.1740-0929.2008.00591.x

[B12] Loh TC Thanh NT Foo HL Bejo MH Kasim A Feeding of different levels of metabolite combinations produced by Lactobacillus plantarum on growth performance, faecal lactic acid bacteria and *Enterobacteriaceae* count, volatile fatty acids and villi height in broilers Anim Sci J 2010 81 205 214 20438502 10.1111/j.1740-0929.2009.00701.x

[B13] Thu TV Loh TC Foo HL Halimantun Y Bejo MH Effects of liquid metabolites combinations by *Lactobacillus plantarum* on growth performance, faeces characteristics, intestinal morphology and diarrhea incidence in postweaning piglets Trop Anim Health Prod 2011 43 69 75 20632092 10.1007/s11250-010-9655-6 PMC2995859

[B14] Thu TV Foo HL TC L Bejo MH Inhibitory activity and organic acid concentration of metabolite combinations produced by various strains of *Lactobacillus plantarum* Afr J Biotechnol 2011 10 1359 1363

[B15] Choe DW Foo HL Loh TC Hair-Bejo M Awis QS Inhibitory property of metabolite combinations produced from Lactobacillus plantarum Strains J Trop Agric Science 2013 36 79 88

[B16] Moghadam MS Foo HL Leow TC Raha AR Loh TC Novel bacteriocinogenic Lactobacillus plantarum strains and their differentiation by sequence analysis of 16S rDNA, 16S-23S and 23S-5S intergenic spacer regions and randomly amplified polymorphic DNA analysis Food Tech Biotechnol 2010 48 476 483

[B17] Thanh NT Loh TC Foo HL Bejo MH Azhar K Inhibitory activity of metabolites produced by strains of *Lactobacillus plantarum* isolated form Malaysian fermented food International Journal of Probiotics and Prebiotics 2010 5 37 44

[B18] Foo HL Loh TC Law FL Lim YZ Kufli CN Gulam R Effects of feeding *Lactobacillus plantarum* I-UL4 isolated from Malaysian Tempeh on growth performance, faecal flora and lactic acid bacteria and plasma cholesterol concentrations in postweaning rats Food Sci Biotechnol 2003 4 403 408

[B19] Choe DW Loh TC Foo HL Hair-Bejo M Awis QS The egg production performance, faecal pH and microbial population, small intestinal morphology, plasma and yolk cholesterol in laying hens fed with liquid metabolites produced by *Lactobacillus plantarum* Strains Br Poult Sci 2012 1 106 115 22404811 10.1080/00071668.2012.659653

[B20] Loh TC Chong SW Foo HL Law FL Effects on growth performance, faecal microflora and plasma cholesterol after feeding with spraydried metabolite in postweaning rats Czech Journal of Animal Science 2009 54 10 16

[B21] Thanh NT Loh TC Foo HL Bejo MH Azhar K Effects of feeding metabolite combinations produced by *Lactobacillus plantarum* on growth performance, faecal microflora population, small intestine villus height and faecal volatile fatty acids in broilers Br Poult Sci 2009 50 298 306 19637029 10.1080/00071660902873947

[B22] SAS Institute Inc SAS User Guide 1998 Cary, NC: Statistic SAS Institute Inc

[B23] Panda AK Reddy MR Rama Rao SV Phaharaj NK Production performance, serum/yolk cholesterol and immune competence of White Leghorn layers as influenced by dietary supplementation with probiotic Trop Anim Health Prod 2003 35 85 94 12636363 10.1023/a:1022036023325

[B24] Xu CL Ji C Ma Q Hao K Jin ZY Li K Effects of a dried *Bacillus subtilis* culture on egg quality Poult Sci 2006 85 364 368 16523640 10.1093/ps/85.2.364

[B25] Mahdavi AH Rahman AR Pourreza J Effect of probiotic supplements on egg quality and laying hen's performance Int J Poult Sci 2005 4 488 492

[B26] Aghaii A Chaji M Mohammad AT Sari M The effect of probiotic supplementation on production performance, egg quality and serum and egg chemical composition of laying hens J Anim Vet Adv 2010 9 2774 2777

[B27] Samli HE Agma A Senkoylu N Effects of storage time and temperature on egg quality in old laying hens J Appl Poult Res 2005 14 548 553

[B28] Dibner JJ Buttin P Use of organic acids as a model to study the impact of gut microflora on nutrition and metabolism J Appl Poult Res 2002 11 453 463

[B29] Ignatova M Sredkova V Marasheva V Effect of dietary inclusion of probiotic on chickens performance and some blood indices Anim Biotechnol 2009 25 1079 1085

[B30] Drider D Fimland G Héchard Y McMullen L Prévost H The continuing story of class IIa bacteriocins Microbiol Mol Biol Rev 2006 70 564 582 16760314 10.1128/MMBR.00016-05 PMC1489543

[B31] Luders T Birkemo GA Fimland G Nissen-Meyer J Nes IF Strong synergy between a eukaryotic antimicrobial peptide and bacteriocins from lactic acid bacteria Applied Environmental and Microbiology 2003 69 1797 1799 10.1128/AEM.69.3.1797-1799.2003 PMC150079 12620872

[B32] Lee D Jang S Baek E Kim M Lee K Lactic acid bacteria affect serum cholesterol levels, harmful fecal enzyme activity and fecal water content Lipids Health and Diseases 2009 24 1 8 10.1186/1476-511X-8-21 PMC2707375 19515264

[B33] Loh TC Foo HL Tan SH Goh YM Shukriyah MH Kufli CN Effects of fermented products on performance, faecal pH, enterobacteriaceae and lactic acid bacteria counts and interrelationships and plasma cholesterol concentration in rats Journal of Animal Feed and Sciences 2003 12 625 636

[B34] Loh TC Thanh NT Foo HL Bejo MH Kasim A **Effects of Feeding metabolite combinations from **** *Lactobacillus plantarum * ****on plasma and breast meat lipids in broiler chickens** Brazillian Journal of Poutry Science 2013 15 307 316

[B35] Thu TV Foo HL Loh TC Bejo MH **Effects of metabolite combinations produced by **** *Lactobacillus plantarum * ****on plasma cholesterol and fatty acids in piglets** American Journal of Animal and Veterinary Science 2010 5 233 236

[B36] Li L Xu CL Ji C Ma Q Hao K Jin ZY Li K Effect of a dried *Bacillus subtilis* culture on egg quality Poult Sci 2006 85 364 368 16523640 10.1093/ps/85.2.364

[B37] Ansorena D Astiasaran I Effect of storage and packaging on fatty acid composition and oxidation in dry fermented sausages made with added olive oil and antioxidants Meat Sci 2004 67 237 244 22061319 10.1016/j.meatsci.2003.10.011

[B38] Mozaffarian D Does α-linolenic acid intake reduce the risk of coronary heart disease? A review of the evidence Alternative Therapies in Health and Medicine 2005 11 24 30 15945135

[B39] Kato T Hancock RL Mohammadpour H McGregor B Manalo P Khaiboullina S Hall MR Pardini L Pardini RS Influence of omega-3 fatty acids on the growth of human colon carcinoma in nude mice Cancer Lett 2002 187 169 177 12359365 10.1016/s0304-3835(02)00432-9

[B40] Leshkanich CO Noble RC Manipulation of the *n*-3 polyunsaturated fatty acid composition of avian eggs and meat Journal of World Poultry Science 1997 53 155 164

[B41] Rolfe RD The role of probiotic cultures in the control of gastrointestinal health J Nutr 2000 130 396 402 10.1093/jn/130.2.396S 10721914

[B42] Ricke SC Perspective on the use of organic acids and short chain fatty acids as antimicrobial Poult Sci 2003 82 632 639 12710485 10.1093/ps/82.4.632

[B43] Immerseel VF De Buck J Pasmans F Velge P Bottreau E Fievez V Haesebrouck F Ducatelle R Invasion of Salmonella enteritidis in avian intestinal epithelial cells in vitro is influenced by short-chain fatty acids Int J Food Microbiol 2003 85 237 248 12878382 10.1016/s0168-1605(02)00542-1

